# Memory of Ensemble Representation Was Independent of Attention

**DOI:** 10.3389/fpsyg.2019.00228

**Published:** 2019-02-08

**Authors:** Shenli Peng, BeiBei Kuang, Ping Hu

**Affiliations:** Department of Psychology, Renmin University of China, Beijing, China

**Keywords:** ensemble coding, ensemble properties, working memory, attention, dual-task paradigm

## Abstract

The hierarchical view of working memory suggested that object ensemble could also be stored into working memory by treating ensemble properties as single “unit.” However, it remains unclear whether ensemble representation in working memory is vulnerable to attention demanding. The present study designed a dual-task paradigm constituting of a memory retaining task and an attention-demanding arrow flanker task. Participants were firstly presented an array (4 or 9) of facial images with neutral expressions and then shown a left- or right-orientated arrow surrounded by four congruent or incongruent oriented arrows or short lines. Participants judged the orientation of the target arrow and then indicated whether a probe facial image was present or absent in the preceding facial array. The probe face consisted of four conditions: (1) a morphed average face of prior face set, (2) a morphed average face of another face set, (3) an exemplar face of prior set, and (4) an exemplar face of another face set. Results confirmed that participants implicitly coded the average facial image of preceding set and retained in working memory. More importantly, the memory representation of ensemble property (e.g., average facial identity) was independent of flanker type. In sum, this study provided further evidence of the hierarchical view of working memory and suggested that attention was not a pre-requisite for the retaining of ensemble properties in working memory.

## Introduction

A number of previous studies have demonstrated that people can automatically extract summary statistics (mean, variability, etc.) from abundant of stimuli, with a surprising precision (Alvarez, 2011; Whitney and Leib, 2018). In a seminal study, Ariely (2001) presented observers with sets of dots in different size and then asked them to indicate whether a single dot presented subsequently was larger or smaller than the mean size of preceding set. Results showed that observers could accurately compute the mean size of a prior dot set while retained little information about individual set members, and this visual averaging ability was not affected by set size. Further, Haberman and Whitney (2007, 2009) demonstrated that ensemble coding also occurred for faces—observers could rapidly extract mean emotional valence of a set of facial expressions, even in a glance (50 or 100 ms). In addition to the universality of object ensembles in our daily life, the rapid course of ensemble coding frees people from the working memory and attentional capacity limitation in representing objects (Luck and Vogel, 1997; Alvarez and Cavanagh, 2004). In other words, puzzle derived from the evidenced working memory capacity limitation and the fact that we can easily map the substantial world have been resolved through the ensemble coding (Lanzoni et al., 2014).

Working memory refers to the temporal manipulation and storage of input stimuli for complex cognitive processing such as learning and reasoning, with a limited capacity (Baddeley and Hitch, 1974; Luck and Vogel, 2013). Among various types of tasks used for measuring working capacity, the change detection task (CDT) was the most popular one. The CDT was firstly proposed by Luck and Vogel (1997) to examine working memory capacity. Usually, during the task, participants were firstly presented with an array of stimuli (e.g. colored squares) for several hundred milliseconds (stimulus array). After a 1s delay period, a probe array constituting of either a single stimulus or the same number of stimuli of the stimulus array was presented and participants had to make a key press to indicate whether there was change between the probe array and stimulus array. Results from CDT studies have confirmed that people have a limited working memory capacity about three to four items in general, and this conclusion was also supported by evidence from neuroelectrophysiological studies (Vogel and Machizawa, 2004; Fukuda et al., 2010; Luck and Vogel, 2013).

As many researchers have stated that the existence of ensemble coding has been broadening our knowledge of visual processing, as well as breaking the limitation of working memory capacity, the relationship between ensemble coding and working memory has been explored by many studies (Brady and Alvarez, 2011; Epstein and Emmanouil, 2017; Nie et al., 2017). In particular, Brady and Alvarez (2011) proposed a hierarchical view of working memory, of which the core theme was that individuals are capable of encoding the gist information of group stimuli (e.g., mean size of an array circles), as well as the exemplar information of the stimulus set. Given the hierarchically structured visual environments around us, this view seems to be adaptive. The hierarchical view has been tested empirically in the ensemble coding field to date (e.g., Brady and Alvarez, 2011, 2015; Im and Chong, 2014; Walker and Vul, 2014). Additionally, by showing the fixed capacity of ensemble coding, several studies (Attarha et al., 2014; Luo and Zhao, 2018) suggested that ensemble representations, like individual items, functioned as “unit” of working memory.

The first aim of this study was to verify whether ensemble properties can be stored as a “unit” in working memory or not, which has been explored in previous studies (e.g., Im and Chong, 2014). Not limited to it, there was also a study (Beaufore, 2017) that explored whether the encoding of ensemble properties was affected or not by global-local attention. In his unpublished thesis, following the CDT procedure, Beaufore (2017) presented participants three squares with either all were bold (global attention) or just one was bold (local attention). Participants were instructed to direct their attention to the bold square(s). Different color was then filled in each square for about 50 ms. After a 200 ms mask display, participants had to indicate the color of the probe square which was cued with a bold frame. In the first study employing a forced choice method, the results showed that there was no difference between the two attention conditions in terms of participants’ response toward mean color. In the second study, the author changed the response format to a continuous report, and the results indicated again no difference between the two conditions, though the responses of the two conditions demonstrating response trend shifting toward ensemble mean. Together, Beaufore (2017) suggested that attention did not moderate the encoding of ensemble properties.

Though needed further test, one might assert that ensemble representation could function as a single “object” in working memory based on the above-reviewed research. Another question was that though it has been explored that the encoding of ensemble statistics in working memory was not modulated by attention (Beaufore, 2017), little is known whether the retaining of ensemble representations could be or not as successful as individual items in working memory and whether it was modulated or not by attention. This formed the second aim of the current study, referring to whether the retaining of ensemble representations that stored in working memory was disturbed by an attention-demanding task or not. To achieve it, a dual-task paradigm consisted of an implicit ensemble coding task and an arrow flanker task was employed.

Implicit ensemble coding task was proposed by de Fockert and Wolfenstein (2009) and widely used for investigations of ensemble coding (Neumann et al., 2013; Rhodes et al., 2015, 2017). The procedure of the implicit ensemble coding task, also called explicit individual member memory task, was similar to that of the CDT. During the task, following a brief fixation display (around 200 ms), participants were presented with an array of different face images (memory array). After a brief exposure, a single probe face was presented on the screen, and participants had to indicate whether the probe facial image presented or not in the preceding multiple faces set (test array). The probe face in this task consisted of four different types, including (1) a morphed average of the prior faces set (matching average), (2) a morphed average of another set of faces (nonmatching average), (3) an exemplar of the prior faces set (matching member), and (4) an exemplar of another set of faces (nonmatching member). Participants in this task were explicitly asked to remember all the stimulus faces in the memory array (without asking to compute the mean face of the faces set). Originally, “present” responses toward four different types of probe faces were collected as dependent variables for statistical analysis (de Fockert and Wolfenstein, 2009; Kramer et al., 2015). However, increasingly, studies have recently employed endorsement scores, an unbiased index, as dependent variable (Rhodes et al., 2015, 2017; Neumann et al., 2017). The endorsement scores were calculated by subtracting the “present” responses of nonmatching conditions from the “present” responses of matching conditions for both set average and exemplar.

The biggest difference between the ensemble coding task and the CDT was that, relative to the CDT, there was no blank period in the ensemble coding paradigm. Thus, this task was rather a perceptual task than a working memory. In the current study, we adapted this task by adding a second task (arrow flanker task), which could be seen as a substitute of the blank display. By doing this, we could firstly test whether the ensemble representations could be “stored” in the working memory through a real working memory procedure (the adapted ensemble coding is actually a memory retaining task in the current study) and secondly explore whether these “stored” units could be successfully retained and retrieved under the interference of an attention-demanding task, specifically, an arrow flanker task in this study.

The flanker tasks (Eriksen and Eriksen, 1974, e.g., arrows) have been well tested to be attention-demanding (Fan et al., 2002) and be used in dual-task paradigm (Cosman and Vecera, 2009; Kim et al., 2017; Wendt et al., 2017). Arrow flanker task requires participants to focus on the target arrow while simultaneously ignoring competing information from distracting stimuli, which referred to selective attention. Usually, there are three types of conditions based on the congruency relationship between target stimulus and distracting stimuli: (1) congruent condition, in which the target arrow is surrounded by an array of arrow with the same orientation, (2) neutral condition, in which the target arrow is surrounded by an array of short straight line, and (3) incongruent condition, in which the target arrow is surrounded by an array of arrow with the adverse orientation relative to the orientation of target arrow. Among above three conditions, slower RT occurs in incongruent condition than in congruent condition, referring to a pattern of attentional conflict effect (Verbruggen et al., 2006). In the present study, we adopted the arrow flanker task as a secondary task to explore the effect of attentional cost on the memory of ensemble properties (e.g., average identity).

## Materials and Methods

### Participants

A total of 39 (11 males) students in Renmin University of China (RUC), who had normal or corrected-to-normal vision, participated in this study. Their average age was 20.74 (*SD* = 2.84) years old, ranging from 17 to 27 years old. This study was approved by the Ethical Committee of the Department of Psychology, RUC. All participants were treated in accordance with the APA’s guidelines. Written consent was informed and obtained.

### Apparatus and Stimuli

The experimental procedure was written and run by E-prime 2.0 and presented on 1,024 × 768 px DELL screen (23.8 inch), and the distance between participants and the screen was about 60 cm. Stimulus faces in this study were 40 male images collected from the native Chinese Affective Picture System (CAPS, Gong et al., 2011). All face stimuli were Chinese with neutral expression, without beards, glasses, or other distinctive facial attributes. Following the morphing procedure used by de Fockert and Wolfenstein (2009), for set size 4 condition, a total of 10 morphed faces, each based on 4 original faces, were created, and for set size 9 condition, a total of 10 morphed faces, each based on 9 original faces, were created. We then improved the fuzzy degree of each original face using the Adobe Photoshop 7.0 to make it more blurred to distinguish. Facial image in set size 4 condition was 180 × 220 px, while in set size 9 condition was 170 × 210 px.

### Procedure

**Figure 1:**
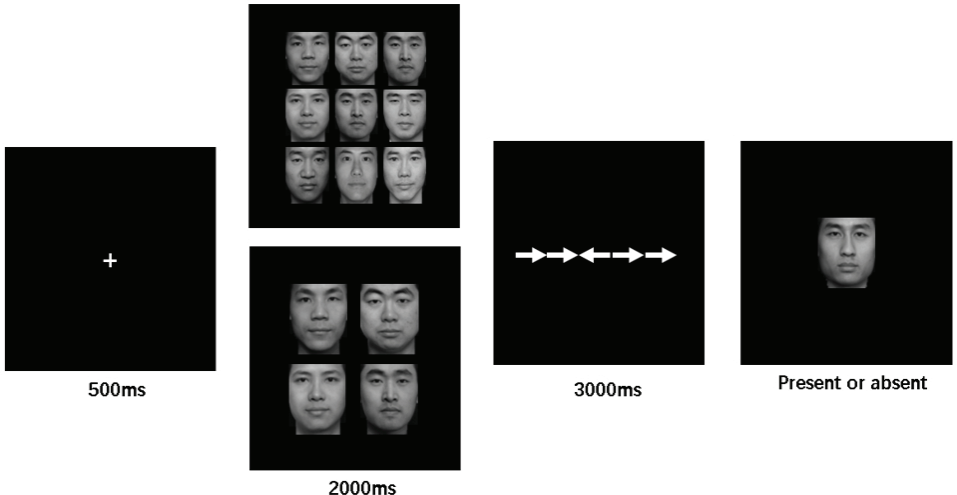
Sequence of display. An array of neutral face images (4/9) presented after fixation and followed by the secondary arrow flanker task. Upon response, a test face presented, and participants required to accomplish the present/absent identification task.

As shown in Figure 1, a trial started with a white fixation cross against the black screen, which was presented in the center for 500 ms. Subsequently, the stimulus display consisted of 4 or 9 neutral face images was presented for 2000 ms and then disappeared. This was followed by a flanker task display, in which a total of five white arrows presented horizontally in the center of the screen. Participants in this display were instructed to make a fast and accurate response to indicate which side the arrow in the center (target) oriented (left or right), while ignoring the distractions from the surrounding arrows. Participants have a maximum of 3,000 ms to respond. After response or 3,000 ms, a test face image flashed on the screen. Here, participants were required to make a second response to decide whether the test face image was present or absent in the prior faces set. The test face image remained on the screen until a response was made, and the inter-trial interval was 1,000 ms. For the arrow flanker task, pressing “f” with left index finger indicated the target arrow was left-oriented, while pressing “j” with right index finger indicated a right orientation; for the ensemble coding task, “f” referred to *present* response, while “j” referred to *absent* response.

There were three conditions of flanker (congruent vs. incongruent vs. neutral) × two kinds of set size (4 vs. 9) × four types of test faces (matching average vs. nonmatching average vs. matching exemplar vs. nonmatching exemplar), making 24 conditions in total. Two types of set size were counterbalanced between block, while the remaining conditions were mixed within each block. Each participant completed eight blocks (60 trials per block).

### Data Analysis

To explore the effect of attention on the retaining of ensemble properties in working memory, we have conducted several ANOVAs based on different dependent variables. First, we chose the “present” responses as the dependent variable as in de Fockert and Wolfenstein’s (2009) study. Specifically, a Flanker type (congruent vs. neutral vs. incongruent) × Set size (4 vs. 9) × Matching (matching vs. nonmatching) × Morph (morph vs. member) repeated ANOVA on “present” responses was conducted. Second, following Rhodes et al. (2015), we employed endorsement scores as an unbiased index of recognition performance in memory retaining task. Based on it, we conducted a Flanker types (congruent vs. neutral vs. incongruent) × Set size (4 vs. 9) × Test type (set average vs. exemplar) repeated ANOVA on endorsement scores. Before the main analysis, we have done some preliminary analysis on the raw data of the flanker task to ensure the validity of the task design in the current study.

## Results

### Preliminary Analysis

Preliminary analysis was conducted on the flanker task firstly to confirm its validity. Trials that the RT was faster than 200 ms or out of the 3 standard deviation of the mean RT were excluded, remaining 97.7% of trials that could be enrolled into the further analysis. Table 1 showed descriptive statistics for the flanker task. For ACC, single-way ANOVA uncovered a main effect of flanker type (F(2, 233) = 9.41, *p* < 0.001), which was driven by the fact that participants performed worse in incongruent than they did in congruent condition (*t* = −0.03, *p* < 0.001) and neutral condition (t = −0.02, *p* = 0.001), with the latter two had no difference (*p* = 0.55). For RT, single-way ANOVA indicated significant differences among three conditions (*F* (2, 233) = 4.73, *p* = 0.011). Post-hoc tests further unfold that average RT in incongruent condition was significantly slower than in both congruent (*t* = 46.88, *p* = 0.028) and neutral conditions (*t* = 62.01, *p* = 0.004), while there was no difference between congruent and neutral conditions regarding to the average RT (*p* = 0.47). In sum, the raw data analysis demonstrated a typical flanker interference effect, that is, participants performed much worse and slower in incongruent condition than they did in congruent and neutral conditions. The results of preliminary analysis confirmed the validity of flanker task as an attention-demanding task in the current study.

**Table 1:** Descriptive statistics for the flanker task (mean ± *SD*).

Conditions	Congruent	Neutral	Incongruent
ACC	0.998 ± 0.001	0.994 ± 0.002	0.972 ± 0.010
RT	642.25 ± 89.33	627.13 ± 83.22	689.13 ± 94.67

### Main Analysis: “Present” Responses as Dependent Variable

**Figure 2:**
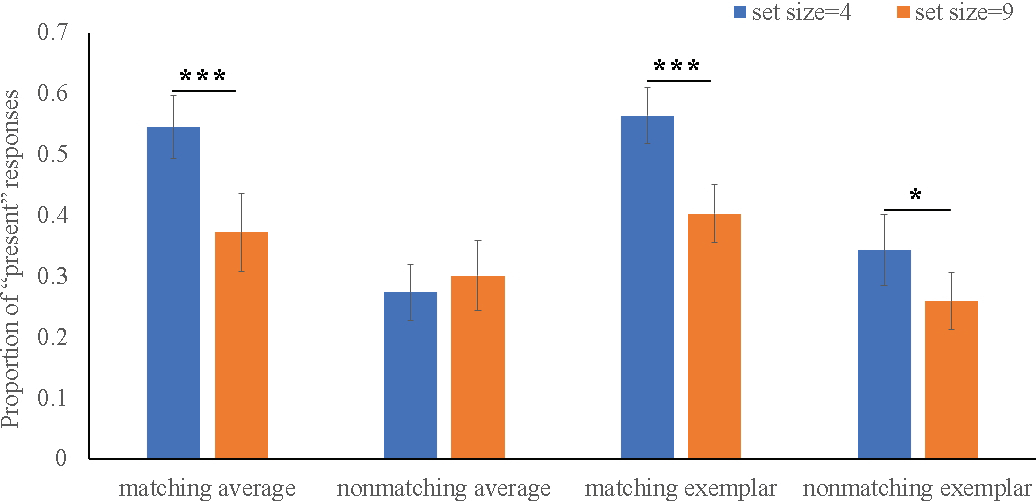
Four-way interaction on average “present” response. Error bars indicate SEM (standard error of the mean). *p < .05, **p < .01, ***p < .001.

As shown in Figure 2, results of Flanker type × Set size × Matching × Morph four-way repeated ANOVA on the “present” responses found main effects of Set size (*F* (1, 38) = 34.04, *p* < 0.001, *η^2^* = 0.67) and Matching factors (*F* (1, 38) = 47.64, *p* < 0.001, *η^2^* = 0.74). For two-way interaction, we found a significant Set size × Matching interaction, *F* (1, 38) = 37.68, *p* < 0.001, *η^2^* = 0.69, showing participants made more “present” responses to matching probe faces when set size was 4 (*M* = 0.56) compared to when set size was 9 (*M* = 0.39), *Differ* = 0.17, *p* < 0.001. For three-way interaction, we also revealed a significant Set size × Matching × Morph interaction, *F* (1, 38) = 4.99, *p* = 0.04, *η^2^* = 0.23, which was driven by the fact that, comparing with set size 9 condition, participants performed more “present” responses to both for matching average and matching exemplar test face in set size 4 condition.

### Main Analysis: Endorsement Scores as Dependent Variable

**Figure 3:**
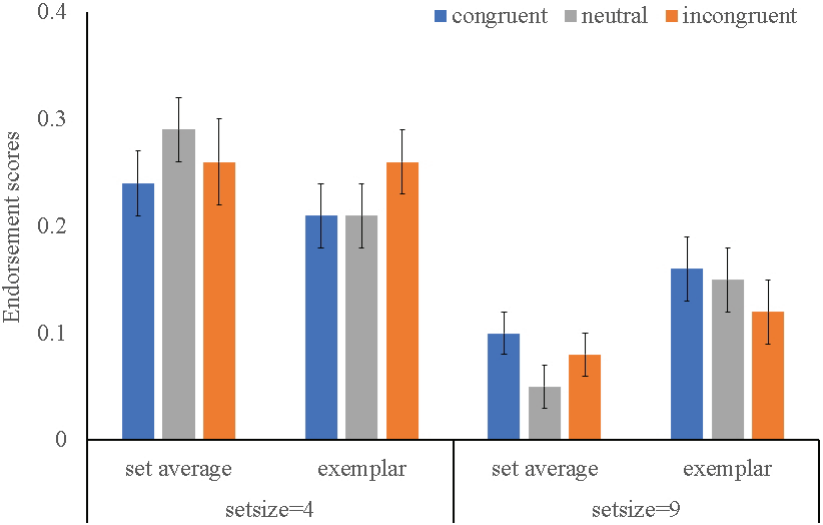
Mean endorsement scores for the set average and exemplar in two set size conditions. Error bars indicate one standard error about the mean.

To note, four participants were excluded from this analysis, for that their endorsement scores of set average in set size 4 conditions were negative values, which indicated they had no discriminability of set average. Results of the Flanker type × Set size × Test type three-way repeated ANOVA on the endorsement scores were shown in Figure 3. A main effect of set size was found, *F* (1, 35) = 83.81, *p* < 0.001, *η^2^* = 0.72. For interactions, only a two-way interaction between Set size and Test type was found significant, *F* (2, 34) = 31.12, *p* < 0.001, *η^2^* = 0.49, which was driven by the fact that participants displayed greater endorsement of set average (*M* = 0.32) than exemplar (*M* = 0.27) in set size 4 condition, while it was reverse in set size 9 condition (*M* = 0.08, *M* = 0.16, respectively). No interactive effect relating to Flanker type was found, except a near significant four-way interactive effect (*F* (1, 38) = 3.41, *p* = 0.06, *η^2^* = 0.30, post-hoc test revealed there was no any effect relating to Flanker type). In sum, analysis on endorsement scores yielded the same outcomes as the analysis on “present” responses, showing that performance of ensemble coding of multiple face identities was independent of attention.

## Discussion

In the present study, we adopted a dual-task paradigm consisted of a memory retaining task and a classical arrow flanker task to investigate whether or not the retaining of ensemble properties in working memory was independent of attention. Consistent with previous studies (Fan et al., 2002; Verbruggen et al., 2006), the results found that participants did show attentional conflict effect during the flanker task, with the lowest ACC and slower RT in incongruent condition than those in congruent and neutral condition. This confirmed the validity of the flanker task used in the current study. More important, the retaining performance of ensemble properties was not modulated by the flanker type, supporting the idea that attention was not a pre-requisite for the implicit maintenance of mean identity for a set of faces.

Set size effect was found consistently when dependent variable was “present” responses or endorsement scores in the present study. This result was in line with past research (Neumann et al., 2017) using implicit ensemble coding task, demonstrating reduced strength of the ensemble coding with increased set size. We suspected that the set size effect in ensemble coding could be attributed to the increasing heterogeneity with the introduction of new faces, suggesting a possible capacity limit of ensemble coding of face identities (Utochkin, 2015; Luo and Zhao, 2018). To note, it seemed that there was a discrepancy between ensemble coding of face-specific stimuli (e.g., identity) and object specific stimuli (e.g., size), as previous studies showing that the strength of ensemble perception was improved with increased set size (Ariely, 2001; Robitaille and Harris, 2011). There were two reasons that we thought were responsible for this discrepancy. The first reason might be that an increment of set size resulted in different outcomes; particularly, for face-specific stimulus, increased set size meant increased heterogeneity, while for object-specific stimulus, increased set size usually produced by repeating set items (e.g., Ariely). The second reason for this dissociation, as suggested by Sama (2017), could be the distinct neural processing network underlying these two kinds of visual stimulus (Duchaine and Nakayama, 2005).

The null effect of flanker types on “present” response we found in the current study suggested that the retaining of ensemble properties (e.g., mean identity) was unaffected by attention. Though there was a marginal significant four-way interactive effect, a risky post-hoc analysis found that the set size effect (larger proportions of matching average and matching exemplar in set size 4 condition than in set size 9 condition) was consistent across flanker types, and the interactive effect was driven by the fact that the set size effect was inconsistent for nonmatching exemplar. This might attribute to the less sensitivity to the difference between morphed average faces of a prior set and another set, for their greater similarities in low-level facial features, such as contour and skin texture. The above result was repeated when we employed endorsement scores as dependent variable. As Rhodes et al. (2015) declaimed, endorsement score was an unbiased recognition index for both set average and exemplar in the ensemble coding task that could prevent the contamination from low-level image properties (e.g., smother skin texture on averages than exemplars). Thus, it is reasonable to suppose that the outcomes derived from the analysis of unbiased endorsement score should be more reliable than of the “present” responses, e.g., the inconsistent set size effect for nonmatching exemplar in the analysis of the “present” responses. Analysis based on the endorsement scores again uncovered that the flanker type did not affect the retaining of average identities. Taken together, whether participants were enrolled into a congruent or incongruent flanker task has no impairment effect on the maintenance of ensemble properties in working memory.

Hierarchical view of working memory proposed that both individual item and ensemble properties could be encoded into working memory, in particular, ensemble properties of a set of similar stimuli could be stored as “units” in working memory for higher cognitive process. This study was in line with the hierarchical view and demonstrated that average identities could be encoded into working memory. Moreover, since there was no research investigating whether or not the retaining of ensemble properties was successful as individual items, the current research acted as the first one to explore this issue. Based on the present results, our study on one hand provided supporting evidence for the hierarchical view and on the other hand expanded the hierarchical view by demonstrating that ensemble properties could be retained well as individual items. The current results that ensemble properties could be encoded and retained well in working memory were in accordance with the reverse hierarchy theory (Hochstein and Ahissar, 2002; Hochstein, et al., 2015). Hochstein and colleagues proposed that processing of ensemble properties was a first-order percept with global attention, which was of importance in overcoming the boundedness of local attention to individual object and the limited capacity of object perception. In other words, encoding and retaining of ensemble properties draw an intact picture of the world, by providing analysis of sets of similar elements (Hochstein et al., 2015). Additionally, Hochstein et al. (2015) claimed this statistic property (e.g., mean) processing occurred rapidly without depending on conscious perception of local details and functioned much in other cognitive processing, such detection of salient deviants.

Given numerous studies have tested that encoding of ensemble properties occurred without any attentional resources (Chong and Treisman, 2003, 2005; Alvarez and Oliva, 2008; Chong et al., 2008; Whitney and Levi, 2011; Bronfman et al., 2014; Beaufore, 2017), the present study provided first-hand empirical evidence that attention was not a pre-requisite of the retaining and retrieval of ensemble properties. Though it was in line with the well-accepted idea that visual system provided ensemble properties with a parallel pathway rather than a serial pathway, we should keep prudent in drawing an absolute conclusion that memory of ensemble properties was not affected by attentional resources at all. One major reason was that we failed to manipulate the number of ensembles in the present study. That was, however the set size was (4 or 9), participants covertly extracted only one average identity of the whole display and retained it in working memory. Theoretically, we were unable to dismiss this possibility, since some recent studies have demonstrated that ensemble coding had limited capacity (Attarha et al., 2014; Im and Chong, 2014; Luo and Zhao, 2018). However, the adapted implicit ensemble coding task in this study was different from the direct ensemble coding task used in previous studies. Additionally, the comparable proportions of “present” responses of average and exemplar, as well as endorsement scores of set average and exemplar between the current study and previous studies (e.g., de Fockert and Wolfenstein, 2009), indicated that it was impossible that retaining performance of even one ensemble in this study would reach a ceiling effect. In sum, further studies manipulating the number of ensembles were in need to confirm the present conclusion that attention was not a pre-requisite for the memory of ensemble properties.

Several limitations should be noted. First, for better presentation, we adopted different size of images in the two set size conditions. One might suspect that the different image size could affect the recognition of face image. We argued that the slightly different size (180 × 220 vs. 170 × 220) we adopted should not be influential enough, for that 1) current results indicated that the larger proportion of “present” responses in set size 4 than in set size 9 was similar to prior studies (e.g., Neumann et al., 2017), and the explanation was discussed above, instead of the image size; 2) past research (e.g., de Fockert and Wolfenstein, 2009; Neumann et al., 2013) using different image size has well evidenced that the existence of ensemble face coding was not affected by the size of the image. Second, the same response key in the primary and secondary task has different meaning for the participants, which might confuse them and thus affect the final outcomes. However, some participants who were randomly selected as interviewee for the experiment reported little or no confuse relating to the experimental procedure, as well as the response keys. Hence, we thought that the response key contributed little to the current results, while we agreed it is better to use different response keys when the two tasks include different psychological meaning. Third, a possible explanation of the current outcomes someone might indicate was the distinct neural substrate of these two tasks we utilized. Previous studies have shown that processing arrow attribute elicited V1 and V2 region, while face processing recruited FFA and ACC; these might contribute to the null effect of arrow flanker task on the visual averaging or face identity. However, we argued that there were other studies demonstrating that incongruent trials in an arrow flanker task could elicit greater neural activities than congruent and neutral trials in ACC (Fan et al., 2003). Therefore, it was less likely the distinction between arrow flanker task and the memory of face ensemble that is responsible for the current results.

In conclusion, this study was, to the best of our knowledge, the first one exploring the relationship between attention and memory of ensemble properties. The current results found that retaining of ensemble properties was not modulated by flanker type, suggesting that attention was not a pre-requisite for the memory of ensemble properties. Further studies were called for to clarify to what extend did the memory of ensemble properties of multiple ensembles (as multiple “objects”) in working memory is affected by attentional resources. This sheds the light of understanding the capacity limitation of retaining of ensemble properties as “units” in working memory.

## Author Contributions

SP and PH developed the study concept and design. SP and BK collected data and analyzed data. SP drafted the manuscript. SP, BK, and PH revised the draft.

### Conflict of Interest Statement

The authors declare that the research was conducted in the absence of any commercial or financial relationships that could be construed as a potential conflict of interest.
